# Chronic trauma impairs the neural basis of empathy in mothers: Relations to parenting and children’s empathic abilities

**DOI:** 10.1016/j.dcn.2019.100658

**Published:** 2019-05-14

**Authors:** Jonathan Levy, Karen Yirmiya, Abraham Goldstein, Ruth Feldman

**Affiliations:** a Interdisciplinary Center, Herzliya, 46150, Israel; b Department of Psychology and the Gonda Brain Center, Bar-Ilan University, Ramat Gan, 5290002, Israel; c Yale University, Child Study Center, New Haven, CT, 06520, USA

**Keywords:** Trauma, Empathy, Magnetoencephalography, Gamma oscillations, Mother-child synchrony, Longitudinal studies

## Abstract

Early life stress carries long-term negative consequences for children's well-being and maturation of the social brain. Here, we utilize a unique cohort to test its effects on *mothers*' social brain, targeting mothers' neural empathic response in relation to caregiving and child empathic abilities. Mother-child dyads living in a zone of repeated war-related trauma were followed from early childhood and mother-child behavioral synchrony was repeatedly observed. At pre-adolescence(11–13 years) children's empathic abilities were assessed and mothers(N = 88, N = 44 war-exposed) underwent magnetoencephalography(MEG) while exposed to vicarious pain. All mothers showed alpha suppression in sensorimotor regions, indicating automatic response to others' pain. However, trauma-exposed mothers did not exhibit gamma oscillations in viceromotor cortex, a neural marker of mature empathy which utilizes interoceptive mechanisms for higher-order understanding and does not emerge before adulthood. Mother-child synchrony across the first decade predicted mothers' viceromotor gamma, and both synchrony and maternal viceromotor gamma mediated the relations between war-exposure and child empathic abilities, possibly charting a cross-generational pathway from mothers' mature neural empathy to children's empathic capacities. Our findings are first to probe the maternal social brain in adolescence in relation to parenting and underscore the need for targeted interventions to mothers raising children in contexts of chronic stress.

## Highlights

• Trauma-exposed mothers do not exhibit gamma oscillations in viceromotor cortex.
• Viceromotor gamma is a neural marker of mature empathy.
• Maternal viceromotor gamma is predicted by mother-child synchrony.
• Maternal viceromotor gamma predicts adolescents' prosociality.

## 1 Introduction

Early life stress (ELS), chronic stress experienced consistently and unpredictably across the first years of life, has been repeatedly shown to carry long-term negative consequences for children's behavior adaptation, susceptibility to psychopathology, stress reactivity, and prosocial skills, and recent studies have also demonstrated its effects on children's social brain (Pitman et al., 2012; Sandi and Haller, 2015). It has been suggested that ELS shapes child outcomes both via its effects on the developing brain and through its impact on the mother and the quality of caregiving, particularly when mother and child are exposed to the same contextual stressors (Birn et al., 2017; Feldman, 2015a; Silvers et al., 2017). Yet, while extant empirical research has focused on the sequalae of ELS in children, no longitudinal study, to our knowledge, addressed the effects of chronic early stress on the mother's social brain. In the current study, we utilized a unique cohort of mothers and children exposed to repeated war-related trauma who were followed from early childhood to preadolescence and tested how chronic stress impacts the neural basis of empathy in mothers. In addition, we asked whether caregiving patterns experienced over extended periods shape not only the child's but also the mother's brain and whether this maternal neural response, in turn, has implications for the development of children's empathic abilities.

### 1.1 Empathy

Empathy is a key social ability most commonly considered from the dual perspectives of shared affect and cognitive mentalization; the first being more rudimentary and evolutionary-ancient, the second more advanced and human-specific (de Waal and Preston, 2017). The first studies on the neural basis of empathy have mainly addressed empathy to vicarious physical pain, and neural response to others' pain has been tested in children, adolescents, and adults and across methodologies, including functional MRI, EEG, and MEG (de Waal and Preston, 2017; Fan et al., 2011; Lamm et al., 2011). Empathic responses to the pain of conspecifics, a capacity sculpted by a long history of mammalian evolution, is critical for species survival and supports the formation of social bonds and group-living (Dulac et al., 2014; Nowak, 2006). Yet, while rudimentary empathy for others' pain is linked with sensory processing and is observed in rodents (Burkett et al., 2016) and primates (Fraser et al., 2008), empathic response in human adults extends to include higher-order representations that enable interpersonal resonance (Sierksma et al., 2014), differentiation of self from other (Zaki et al., 2016), and cognitive understanding of others' needs and emotions (Shamay-Tsoory et al., 2009). Extant research has shown that various psychiatric disorders are associated with diminished capacity for empathy (Ponnampalam, 2018) and research on the brain basis of empathy indicated impairments in cases of depression (Merkl et al., 2016), conduct disorders (Decety et al., 2013) and PTSD (Eidelman-Rothman et al., 2016), psychopathologies that involve severe disruptions to adaptive social life. Thus, intact and mature functioning of the neural systems that sustain empathy are likely to play a key role in the individual's capacity for social participation and the ability to adequately read and respond to social signals (Bernhardt and Singer, 2012; Keysers and Gazzola, 2007), highlighting the need to study the neural basis of empathy across contexts and conditions. Parenting provides a key context for the experience and practice of empathy (Feldman, 2016, 2017) and studies on the parental brain underscored the importance of brain networks implicated in empathy for the development of parenting and children's social-emotional outcomes (Abraham et al., 2017). It is thus of interest to examine how the experience of parenting in the shadow of continuous trauma impacts the neural basis of empathy in mothers.

The neural substrates of empathy undergo significant maturation throughout life (Decety and Michalska, 2010). In a large developmental magnetoencephalography (MEG) study, we found that whereas children and adolescents exhibit alpha and beta oscillations in sensori-motor cortex (S1) to others' pain, adults additionally recruit gamma-band activity in viceromotor cortex (Levy et al., 2018), implicating higher-order affective representations. Research in humans and animals has shown that gamma-band activity does not emerge before developmental maturity (Chang et al., 2016; Fabrizi et al., 2016; Uhlhaas et al., 2009) and gamma oscillations have been shown to integrate higher-order information in viceromotor regions during pain perception (Nickel et al., 2017; Schulz et al., 2015). It is thus possible that viceromotor gamma may chart a neural marker of maturity that extends from the automatic, sensori-motor based type of empathy observed in mammals and human children to the higher-level empathy observed in human adults. Furthermore, frontal viceromotor regions, including the anterior insula (AI), anterior cingulate cortex (ACC), and orbitofrontal cortex (OFC), have been shown to underpin affect understanding and higher-order representations during empathy to others' pain (Fan et al., 2011; Singer et al., 2004; Zaki and Ochsner, 2012). Such viceromotor mechanisms enable adults to draw upon the experience of one's own bodily milieu (i.e., interoception) for representing others' physical pain or mental states (Feldman Barrett and Simmons, 2015). Similar to empathy, interoception is a fundamental mechanism that is disrupted in various psychiatric conditions (Murphy et al., 2017), indicating that the ability to accurately represent one's body indexes resilience and health (Khalsa et al., 2018) and plays a role in the development of social competencies (Maister et al., 2017; Mundy and Jarrold, 2010).

### 1.2 Mother-child synchrony and empathy

The development of empathy in children is sustained by sensitive parental care, particularly the experience of parent-infant synchrony where the parent and child coordinate their gaze, affective expression, posture, and social communications into a matched dialogue that promotes positive engagement and mutual understanding (Feldman, 2007a). Longitudinal studies following children from infancy to late childhood and adolescence have shown that the experience of synchrony during the first years of life shapes the child's later capacity for empathy (Feldman, 2007b, 2015b). Furthermore, adolescents' neural empathic response to others' pain is predicted by the experience of synchronous parenting across the first decade of life and when synchrony is reduced, for instance, in cases of maternal postpartum depression, the child's neural empathic response is disrupted (Pratt et al., 2016). Notably, synchrony has been linked with gamma rhythms. For instance, in response to attachment stimuli mothers and their 9-year old children synchronize their gamma oscillations in the posterior STS and this neural coupling is predicted by the degree of behavioral synchrony in early childhood (Levy et al., 2017). Similarly, romantic partners display brain-to-brain synchrony of gamma rhythms in temporal regions during naturalistic interaction and such gamma coupling is anchored in moments of behavioral synchrony (Kinreich et al., 2017). Gamma-band activity supports not only higher-order empathy (Levy et al., 2018), but also underlies non-verbal emotional communication (Symons et al., 2016), suggesting that synchronous experiences within the parent-child context may tune the parental brain to the more mature mechanism of viceromotor gamma that sustains higher-order empathy.

### 1.3 The current study

In the current study, we utilized a unique cohort of mothers and children living in a war zone in the south of Israel, an area that has been exposed to continuous war-related trauma for over two decades. This area is located near the Gaza border and its citizens live under continuous terror threat. During the past 12 years, the area has suffered frequent and unpredictable rocket attacks and six military operations involving daily, repeated, and intense missile attacks. Since 2001, dozens of civilians have been killed, more than 2000 injured, and a significant property and infrastructure damage has resulted from these attacks. Citizens live under constant threat; any given moment a siren alert may erupt allowing only a few seconds to enter sheltered spaces before explosion occurs. Such constant stress has caused significant trauma and distress among adults and children (Feldman and Vengrober, 2011).

Mothers and children were followed from early childhood, patterns of mother-child synchrony were repeatedly assessed across childhood, and maternal MEG scanning was conducted at the transition to adolescence (11–13y). Notably, our study offers a unique "natural experiment" on the effects of chronic trauma on parents and children, as all families experienced the same trauma, a rare condition in research on ELS, while individual and relational factors differentiated among families. Also unique is our choice to focus on mothers' social brain in the context of chronic stress, complementing research on the effects of ELS on children's social brain (Sheridan et al., 2018). We assessed maternal brain response to vicarious pain and while we expected alpha suppression in sensory-motor regions in all mothers, consistent with prior EEG/MEG studies (Mu et al., 2008; Whitmarsh et al., 2011), chronic stress was expected to blunt the higher-order expression of viceromotor gamma (hypothesis 1). We also expected that the experience of mother-child synchrony across the first decade of life would provide practice for the higher-order empathy and predict more gamma activity, consistent with parallel findings in children who received synchronous parenting (Levy et al., 2017; Pratt et al., 2018) (hypothesis 2). Finally, we expected that greater maternal viceromotor gamma, indicating greater maternal understanding of others' feelings and mental states, would predict better child prosocial skills, charting a cross-generational pathway from empathy in the maternal brain to the child's empathic capacities as mediated by patterns of mother-child synchrony (hypothesis 3).

## 2 Materials and methods

### 2.1 Participants

**Fig. 1:**
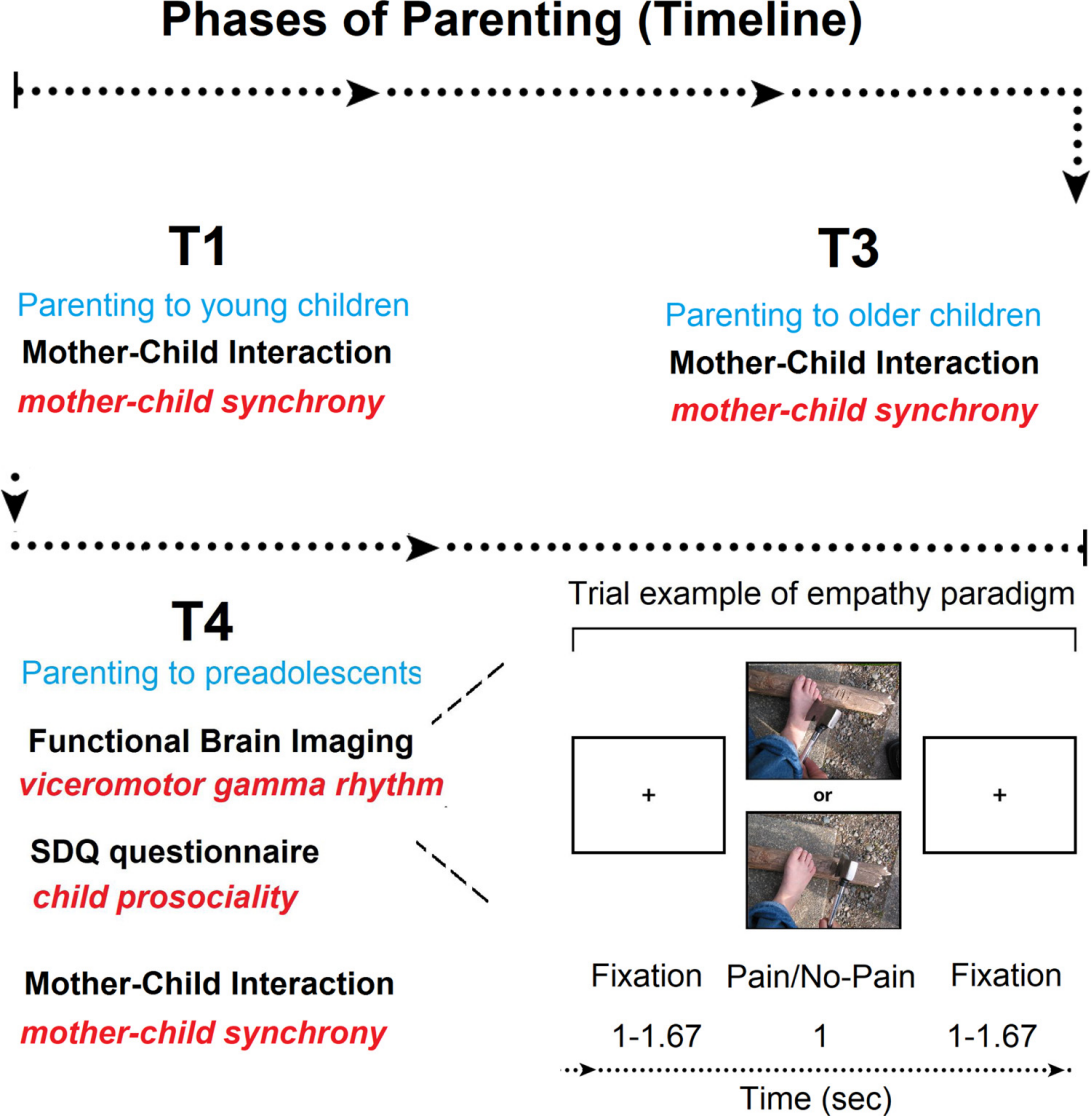
Timeline of the study from T1 to T3 until T4. Blue color refers to the parenting phase, black to the modality/method, and red to the variable of interest. In T4, the functional brain imaging paradigm is illustrated (lower right panel) (For interpretation of the references to colour in this figure legend, the reader is referred to the web version of this article).

Participants were recruited in two groups and observed four times as follows (Fig. 1).

*T1 - Parenting young children*: We recruited 232 mothers to young children (M = 2.76 years, SD = 0.91). The war-exposed group included 148 families living nearby the Gaza border and exposed to continuous and unpredictable rockets and missiles attack for over 20 years. The control group included 84 non-exposed families from comparable towns in the greater Tel-Aviv area matched to exposed group in age, gender, birth order, parental age and education, maternal employment and marital status and screened for other types of trauma.

*T2 - Parenting in middle-childhood: middle childhood*; children underwent psychiatric diagnosis and these data are not used here.

*T3 - Parenting of older children*; we revisited 177 of the mothers, now to older children (M = 9.3 years, SD = 1.41). Attrition was mainly related to inability to locate families or families moving out of Sderot.

*T4 - Parenting preadolescents*: Eighty-eight mothers^1^ participated in MEG scanning (M = 40.61 years, SD = 5.15) and their children were now in preadolescence (M = 11.81 years, SD = 1.24); half of mothers (n = 44) were war-exposed (see Table 1 for demographic comparisons). Taxi transportation was arranged to bring the mothers to the Gonda Brain Research Center at Bar-Ilan University, where they underwent the MEG experiment. Mothers received monetary compensation for their participation. Of the 107 mothers participating in T4, 19 did not complete the MEG experiment: 8 were MEG-incompatible or with poor MEG signal, 5 declined the MEG part, 3 did not complete the paradigm, and 2 were pregnant. Study was approved by the local IRB and written consent was obtained from participants after receiving complete description of procedures. Experiments were performed in accordance with ethical guidelines.

**Table 1:** Group comparisons on demographic variables.

	Control	Exposed	t/χ^2^	Effect size (Cohen's d/ Φ)
**Child Age**	M = 11.57 (SD = 1.18)	M = 11.72 (SD = 1.37)	t_(86)_ = −.53, p = .56	.12
**Mother age**	M = 41.18 (SD = 4.48)	M = 40.05 (SD = 6.05)	t_(86)_ = .99, p = .31	.21
**Mother education** Above high school	36 (83.7%)	29 (67.4%)	χ^2^_(1)_ = 3.09, p = .08	.19
**Maternal marital status** Married	41 (93.2%)	41 (93.2%)	χ^2^_(4)_ = .74, p = .74	.15
**SES (1-9)**	M = 4.65 (SD = 1.37)	M = 4.19 (SD = 1.07)	t_(84)_ = 1.76, p = .08	.37

### 2.2 Severity of trauma exposure

Severity of trauma exposure was evaluated using a questionnaire of life events. Mothers were asked whether someone from their immediate or extended family or a close friend was directly exposed to an act of terror or war-related violence. Out of the 44 war-exposed families, eight families described being exposed to an event of "severe trauma exposure". This meant (a) their home, or the home of a family member, was damaged by a missile attack, (b) a family member or a close friend was injured in an attack; or (c) a family member or a close friend was injured during a military operations in Gaza.

### 2.3 MEG experimental design

*Stimuli.* We programmed and operated the experiment using E-Prime® software (Psychology Software Tools Incorporated). We used two types of stimuli: pain(P) and no-pain(no-P) stimuli (Jackson et al., 2005). All stimuli appeared in uniform size (300 × 225 pixels) at the center of gray background on a 20-inch monitor, at viewing distance of approximately 55 cm. A series of 96 digital color pictures showed limbs (right hands and right feet) in P(48 stimuli) and no-P(48 stimuli), at ratio of 51/49% for legs/hands. The purpose of P stimuli was to elicit empathy for pain, while of no-P stimuli to control for the other parameters induced by the visual stimuli.

*Procedure.* Participants lay in supine position inside the MEG while facing a screen projecting the stimuli. Subjects received instructions to remain relaxed, to not move their limbs, and to watch the stimuli. Experimenters observed their compliance using an infrared camera. As illustrated in Fig. 1 (lower right panel), P and no-P stimuli (in total, 110 trials per experiment) were presented for 1 s each, interleaved by crosshair fixation screens randomly varying in duration between 1 and 1.67 s. Consistent with prior studies (Levy et al., 2016; Pratt et al., 2016; Whitmarsh et al., 2011), in order to maintain attention throughout the session, we randomly inserted attentional filler trials (11% of all trials) by creating a short twisted movement in additional stimuli using a twirl filter (Photoshop, Adobe Systems Inc.). Participants were instructed to press a button when detecting these stimuli and were trained on the task before the session started. We did not include the filler trials in the experimental stimuli database or analyse them.

### 2.4 Social behavior

*Mother-child synchrony* was observed at T1, T3, and T4 in age-related paradigms (T1: free play, T3: discussion paradigms, T4: joint task) and coded with the Coding Interactive Behavior Manual(CIB) (Feldman, 1998). The CIB is a well-validated system for coding social behavior extensively used across cultures and psychiatric conditions from infancy to adulthood (Feldman, 2012). It includes multiple scales coded from 1(low) to 5 (high) which are averaged into theoretically-determined constructs. The final *mother-child synchrony* construct (Halevi et al., 2017) included the following scales from all three stages of the study: dyadic reciprocity, mutual regulation/adaptation, interactive fluency. At T3 and T4 scales of empathy, supportive presence, positive affect, recognition, expansion, containment, and appropriate expression were also added. All variables were averaged, firstly in each time point (Cronbach's α T1 = .93; T3 = .92; T4 = .95), and then across the three time-points, to a single composite mother-child synchrony variable (Cronbach's α = 0.91). All further analyses of mother-child synchrony refer to this longitudinal score.

*Child Prosocial Skills* was examined with the Strengths and Difficulties Questionnaire(SDQ) (Goodman, 1997), a well-validate questionnaire for children aged 4-17. The SDQ contains 25 items that comprise 5 scales of 5 items each, each scored on a 3-point Likert scale, with acceptable test–retest reliability and construct validity (Stone et al., 2010), and correlations with other well-established measures (Goodman and Scott, 1999). The prosocial scale includes items such as: "I try to be nice to other people", "I am helpful if someone is hurt, upset, or feeling ill", (Cronbach's α = 0.66).

### 2.5 MEG recordings and data preprocessing

We recorded ongoing brain activity (sampling rate, 1017 Hz, online 1–400 Hz band-pass filter) using a whole-head 248-channel magnetometer array (4-D Neuroimaging, Magnes® 3600 WH) inside a magnetically shielded room. Reference coils located approximately 30 cm above the head, oriented by the x, y and z axes enabled removal of environmental noise. External noise (e.g, power-line, mechanical vibrations) and heartbeat artifacts were removed from the data using a predesigned algorithm for that purpose (Tal and Abeles, 2013). We analyzed data of 2300 ms epochs including baseline period of 450 ms filtered in the 1–200 Hz range with 10 s padding and then resampled them to 400 Hz. Then, spatial component analysis (ICA) was applied in order to clean eye-blinks, eye movements or any other potential noisy artifacts, and finally, data was inspected visually to reject any remaining artifacted trials.

### 2.6 Spectral and source analyses

We attached five coils to the participant's scalp to record head position relative to the sensor. We performed analyses using MATLAB 11(MathWorks®, Natick, MA, USA) and the FieldTrip software toolbox (Oostenveld et al., 2011). We applied tapers to each time window to compute Time-Frequency Representations(TFRs) of power for each trial and to calculate the Fast Fourier Transform(FFT) for short sliding time-windows. We analysed data in alignment to stimulus onset and then averaged the power estimates across tapers. To probe gamma-frequency power (40–150 Hz), five Slepian multitapers (Percival and Walden, 1993) were applied using fixed window length of 0.2 s, resulting in frequency smoothing of 15 Hz. We obtained induced activity by subtracting evoked-components' power from oscillatory power. Finally, we determined time-frequency windows where the P vs no-P contrast was statistically significant after correcting for multiple comparisons.

For source localization, head shape underwent manual digitization (Polhemus FASTRAK® digitizer), and a single shell brain model was built based on MNI post-puberty template brain (Fonov et al., 2011), which we modified to fit each subject's digitized head shape using SPM8 (Wellcome Department of Imaging Neuroscience, University College London,www.fil.ion.ucl.ac.uk). We then divided the subject's brain volume into a regular grid, obtaining the grid positions by their linear transformation in canonical 1 cm grid. This procedure facilitates group analysis, because it requires no spatial interpolation of the volumes on reconstructed activity. Finally, we used the statistically significant time-frequency windows obtained at the sensor level analyses to proceed with beamforming: For each grid position, we reconstructed spatial filters (Gross et al., 2001) using partial canonical correlations (i.e., PCC), in the aim of optimally passing activity (in that time-frequency window) from the location of interest, while suppressing activity that was not of interest. This step would allow to localize the main cortical source, at the pinpointed (i.e., first sensor level step) time-frequency range, responding to the empathy task. Finally, time series were extracted from the peak group activation coordinates (i.e., same peak location for every participant) by applying a linear constrained minimum variance beamformer (i.e., LCMV), at the same pinpointed frequency range. This second analysis step relies on a different source-localization algorithm, and therefore its output is a validity check to the sensor-level output, as well as to the first source results. By plotting the temporal activation pattern in the cortical coordinates of interest, we would be able to compare it to the time-frequency sensor analysis and to the source localization activation pattern. We were interested to determine the statistically significant time points during which the empathic response was both above baseline and more robust than the control stimuli (i.e., no-Pain).

### 2.7 Statistical analyses

#### 2.7.1 MEG data

Statistical procedures on the MEG data assessed significance of the power values using non-parametric approach (Maris, 2007) which takes the cross-subject variance into account, as this variance is the basis for the width of the randomization distribution. This approach is valuable because it does not make assumptions about underlying distributions and is unaffected by partial dependence between neighbouring time-frequency pixels. Specifically, in the first step of the procedure we computed t-values per subject, channel, frequency, and time, representing the contrast between conditions. Subsequently, we defined the test statistic by pooling the t-values over all participants. Here, we searched time-frequency clusters with effects that were significant at the random effects level after correcting for multiple comparisons along the time and the frequency dimensions. To compute the effect compared to baseline, the first step was replaced by adjusting the effect to the baseline level, and the second step applied a dependent t-test. These procedures would correspond to a fixed-effect statistic; however, to make statistical inferences corresponding to a random effect statistic, we tested the significance of this group-level statistic via a randomization procedure: We randomly multiplied each individual t-value by 1 or −1 and summed it over participants. Multiplying the individual t-value with 1 or −1 corresponds to permuting the original conditions in that subject.

We reiterated this random procedure 1000 times to obtain the randomization distribution for the group-level statistic. For each randomization, we retained only the maximal and the minimal cluster-level test statistic across all clusters, placing them into two histograms that we addressed as maximum/minimum cluster-level test statistic histograms. We then determined, for each cluster from the observed data, the fraction of the maximum/minimum cluster-level test statistic histogram that was greater/smaller than the cluster-level test statistic from the observed cluster. We retained the smaller of the two fractions and divided it by 1000, giving the multiple comparisons corrected significance thresholds for a two-sided test. The proportion of values in the randomization distribution exceeding the test statistic defines the Monte Carlo significance probability, which is also called a *P* value (Maris, 2007). This cluster-based procedure allowed us to obtain a correction for multiple comparisons in all brain analyses.

#### 2.7.2 Behavioral data

Exposure was dummy coded, with the control group given a value of “0” and the exposed group a value of “1”. T-tests were used to compare brain and behavior variables between exposed and controls. Next, Pearson correlations assessed the relationships between study variables. In order to estimate the unique contribution of exposure and maternal behavioral empathy on maternal Gamma activity, a linear regression was conducted with war-exposure and behavioral synchrony as independent variables, and maternal Gamma activity as the dependent variable. Finally, for a comprehensive model of the direct and mediated paths leading from war-exposure to child prosocial skills as mediated by maternal brain activity and mother-child synchrony, we used contemporary practices of the simple linear mediation model by Hayes (Hayes, 2013a). Specifically, Baron and Kenny (Baron and Kenny, 1986) proposed a causal steps approach, in which several regression analyses are conducted and significance of the coefficients is examined at each step in order to test a causal chain of theoretical influences of mediation. Preacher and Hayes (Preacher and Hayes, 2008) have formulated a new approach of mediation following later technological and theoretical developments, that attempt to overcome some of the problems in the multi-stage approach. In general, their approach suggests a way to test the direct mediation without relying on preliminary stages. Therefore, the first stage of the multi-stage approach is permitted and there is no requirement for a significant direct relationship between the dependent and the independent variables. Based on the Preacher and Hayes (Preacher and Hayes, 2008) approach, in the current study, we estimated the conditional effect of the independent variable “exposure” (X) on the outcome variable “child prosociality” (Y), with maternal brain activity and mother-child interaction as mediators (M1, M2). The PROCESS macro for SPSS (v. 2.1.3.2) Model 6 (Hayes, 2013b) was utilized for this analysis. PROCESS employs bootstrapping calculations, a nonparametric resampling procedure, which provides the most powerful and reasonable method of obtaining confidence limits for specific indirect effects (Preacher and Hayes, 2008). For these analyses, bias-corrected standard errors and confidence intervals were generated using 10,000 bootstrapped samples. Mediation is considered present when the confidence interval for the estimation of the indirect effect does not contain zero.

## 3 Results

### 3.1 MEG paradigm findings

To probe our first hypothesis, that is, that chronic stress would blunt the higher-order expression of gamma in empathy-related cerebral regions, we first probed the neural effect of empathy for pain (Pain vs no-Pain) at the whole MEG sensor-array level. The statistical time-frequency maps (0–2 s; 30–150 Hz) in the two groups are represented on Fig. 2, with statistically significant time-frequency patterns *(P*_cluster-cor_ <0.05) in the high gamma range (100–130 Hz) between 1150–1350 ms post stimulus onset in the control group as we have already shown (Levy et al., 2018); yet, the exposed group did not yield that pattern (p > .25). Likewise, in the low-frequencies spectrum (0–2 s; 1–30 Hz) there was a significant *(P*_cluster-cor_ <0.05) suppression across the whole low-frequencies spectrum in both groups, consistent with our prior findings (Levy et al., 2018).

**Fig. 2:**
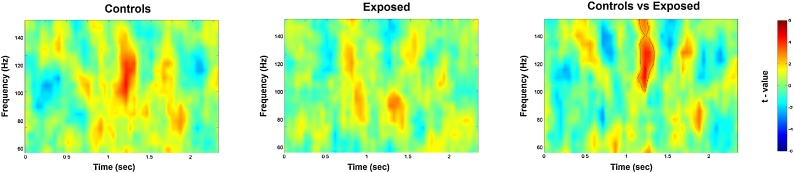
Sensor-level spectral maps conveying empathy for pain. The statistical maps of Pain vs no-Pain stimuli are averaged above all sensors in the two groups (left and middle panels), and in the contrast between the two groups (right panel). The contoured pattern illustrates statistically significant (p _cluster-cor_ < 0.05) time-frequency windows resulting from comparing the two experimental groups.

We then proceeded to test whether this observation is reflected at the between-group level. We found a statistically significant difference *(P*_cluster-cor_<0.01) between the two groups with the contrast yielding similar pattern to that in the controls group (Fig. 2, pattern contoured in black line). By contrast, in the low-frequencies, there was no statistically significant difference (p > .50) between the two groups. Overall these findings indicate that the gamma-band activity pattern is what differentiates the two groups – exposed participants lack it, whereas they yield the typical low-frequency patterns (Levy et al., 2018).

**Fig. 3:**
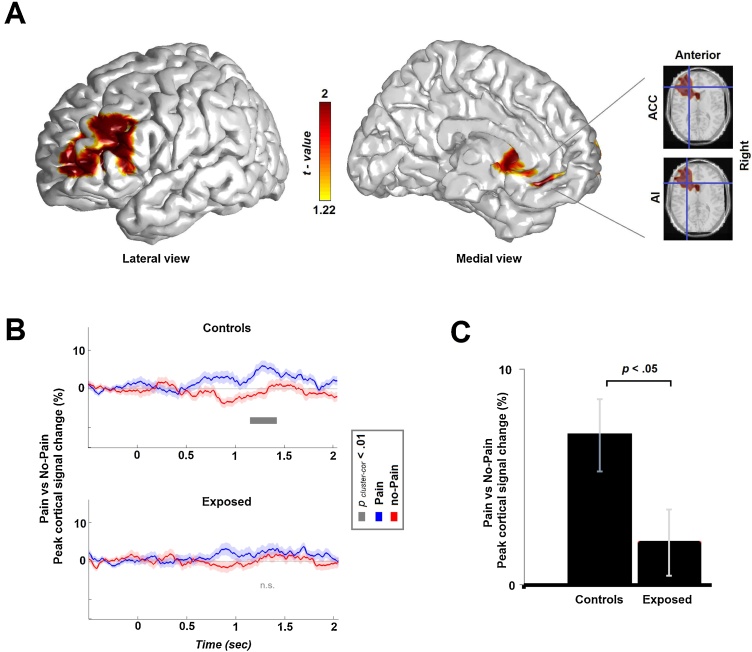
(**A**) Source-level localization of the gamma-band spectral pattern overlaid on MNI template. Color on the template represent peak statistical activity (p _cluster-cor_ < 0.05) in the left-hemisphere, with orientations mentioned (e.g., lateral/medial, right, anterior). Virtual channel was extracted from the peak source location and averaged across the sensor-level frequency band, using the (**B**) LCMV and the (**C**) PCC beamformers. The first enables the representation of time-series in a specific cortical location, whereas the second localizes the source on the time samples of interest.

Next, using PCC beamforming, we conducted source localization on the effect (in the previously pinpointed time-frequency of interest) and found a left-hemispheric peak *(P*_cluster-cor_ < 0.05) activation cluster in the viceromotor(VM) cortex(Fig. 3). The axial insets reveal that beside the cluster peak in the left Medial Frontal Gyrus (t = 3.87; MNI coordinates: −20 44 0, Brodmann area 10), the cluster includes two substrates which are functionally-selective to empathy: (a) the Anterior Cingulate Cortex (t = 3.44; MNI coordinates: −24 34 10, Brodmann area 32) and the (b) Anterior Insula (t = 3.29; MNI coordinates: −30 26 10, Brodmann area 13). A virtual channel analysis on the cluster’s peak confirmed the findings at the sensor level – the effect was significantly above baseline only in the controls group *(P* < 0.05), and significantly more robust *(P* < 0.05) than in the exposed group. To further validate these findings, we conducted a second analysis step: extracting time-series from this cortical location (peak coordinates) by applying a different beamformer technique (i.e., LCMV). Results from this analysis are illustrated in the time-series plots on Fig. 3, and reveal that between 1.16 and 1.41 s, the selective cortical response to empathy was above baseline (i.e., zero) and more robust than the control condition (i.e., no-empathy stimuli). This, however, was the case only in the controls group (*p _cluster-cor_ < 0.01)*, not in the trauma-exposed group, thereby further consolidating the findings from the previous analysis steps. Altogether, analyses support our first hypothesis, that is, that chronic stress may blunt the higher-order empathy expression of gamma cortical activity.

### 3.2 Links to social behavior

**Fig. 4:**
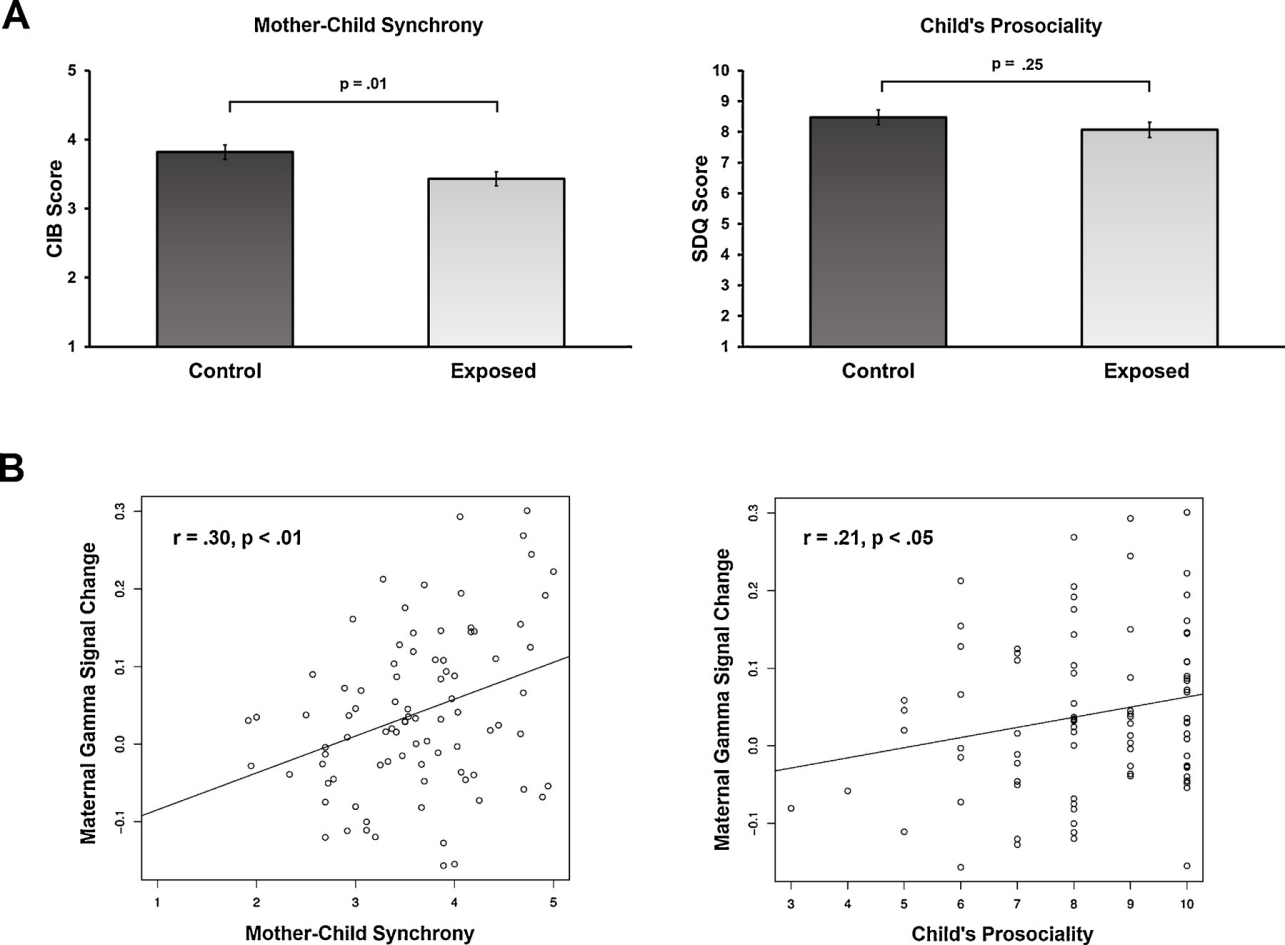
(**A**) Control mothers had significantly higher levels of synchrony behavior with their child compared to mothers in the exposed group (t(86) = 2.58, p = .01, Cohen's d = .56). Children in the exposure group did not differ from control children in their prosocial score (t(86) = 1.16, p = .25, Cohen's d = .25). (**B**) Pearson correlations of cortical gamma signal change (from 0 to 1, with 1 meaning 100% change) with synchrony and prosociality are displayed.

To probe our second hypothesis, that is, that the experience of mother-child synchrony across the first decade of life provides practice for the higher-order empathy and predicts more gamma activity, we first examined group differences in mother-child synchrony and child prosocial skills (Fig. 4). T-tests revealed that exposed mothers had significantly less gamma activity and displayed less synchrony with their children. Exposed and control children did not differ in their prosocial score. In order to evaluate whether the severity of the exposure influenced our results, a MANOVA test was conducted with 'severity of trauma exposure' as an independent variable (control/ mild exposure/ severe exposure) and maternal behavioral synchrony, maternal Gamma activity and child's prosocial score as depended variables. Results indicated a non-significant effect (F_(6,168)_ = 1.75, p = .11), and post-hoc comparisons showed no differences between mothers and children with and without severe exposure (p > .05). Therefore, in the following analyses war-exposure was treated as a dichotomous variable (control vs. exposed). Analysis of the association between social behavior and brain activity demonstrated a significant correlation between mother-child synchrony and gamma activity (Table 2 and Fig. 4). Hierarchical regression predicting maternal gamma-band activity from war-exposure and mother-child synchrony is shown in Table 3. This regression shows that synchrony uniquely predicted gamma above and beyond exposure and the model predicted 11.4% of the variance in gamma. These analyses provide support for our second hypothesis, that is, that the experience of mother-child synchrony predicts more empathy as reflected via cortical gamma activity.

**Table 2:** Pearson correlations of study variables.

Variable	Mother-child synchrony	Gamma activity
Gamma activity	.30**	--
Child prosocial score	−.09	.21*

* p < .05.

** p < .01.

**Table 3:** Hierarchical linear regression predicting Gamma wave activity from exposure to war and mother-child synchrony.

	β	R^2^ Chang	F Change
Predictors			
Exposure	−.23*	.05	4.61*
synchrony	.26*	.06	6.02*

R^2^ Total = .11, *F* (2,85) = 5.45, *p < .05, **p < .01, ***p< .001.

**Fig. 5:**
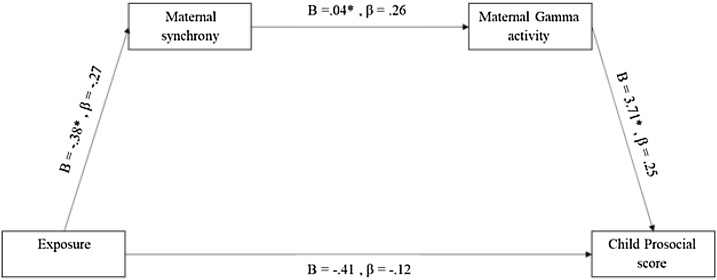
A mediation analysis pointing out the indirect effect of exposure (X) on child prosocial score (Y) through maternal synchrony (M1) and Gamma activity (M2).

To probe our third hypothesis, that is, that the greater maternal gamma activity predicts better child prosocial skills, we explored direct and mediated paths linking exposure to child prosociality, as mediated by synchrony and gamma; we used process modeling outlined by Hayes (2013), using PROCESS macro. War-exposure significantly predicted lower mother-child synchrony (β=−.27, SE = .10, p < .05), such that the percentage is 34% lower among the exposed group. Next, we found that mother-child synchrony had an effect on mothers' gamma activity (β = .26, SE = .11, p < .05); an increase of 1% in mother-child synchrony was associated with an increase of 4% in maternal gamma activity. Finally, maternal gamma activity predicted children' prosociality (β = .25, SE = .11, p < .05), and the percentage of gamma activity among mothers increases the SDQ score for prosociality by 3.71. These trends are illustrated in Fig. 5. These unstandardized indirect effects were computed for each 10,000 bootstrapped samples, and the 95% confidence interval was computed by determining the indirect effects at the 2.5th and 97.5th percentiles. The bootstrapped unstandardized indirect effect was −.02, and the 95% confidence interval ranged from .01 to .16, suggesting that mother-child synchrony and gamma activity mediated the relations between war exposure and child prosociality. The effect is negative, suggesting that exposure to war decreases mother-child synchrony, which in turn increases maternal gamma activity. Maternal Brain activity is positively associated with SDQ prosocial score, i.e., when maternal empathic brain activity increases, children's prosociality increases. Together, these analyses strengthen the validity of our third hypothesis, that is, that the greater maternal gamma activity predicts better child prosocial skills.

## 4 Discussion

Results of the current study demonstrate that raising children in a region of chronic and unpredictable stress takes a toll on the mother's social brain, in addition to the well-documented effects on her caregiving behavior (Halevi et al., 2017; Laor et al., 2001; Leen-Feldner et al., 2013; Lieberman et al., 2005; Pat-Horenczyk et al., 2015; Smith et al., 2001; Ulmer-Yaniv et al., 2018; Yirmiya et al., 2018). Furthermore, our results show a cross-generational, brain-behavior link between activations in the mother's social brain and children's prosocial abilities, charting a trajectory from the maternal brain to the child's social outcome at the transition to adolescence. Specifically, we demonstrate impairments to the neural basis of empathy in mothers living in a zone of war and raising children under chronic stress (hypothesis 1). We further found that such disruptions are shaped by patterns of caregiving over time and have implications for children's prosocial and empathic abilities in early adolescence. Our findings have unique contributions to three lines of research in social neuroscience as well as important implications for clinical practice. First, this is the first study, to our knowledge, to examine the neural basis of general empathy, unrelated to stimuli of the attachment target, in the context of parenting. Second, we show for the first time how patterns of caregiving measured over a decade of parenting shape the maternal social brain (hypothesis 2). Although studies are beginning to address how maturation of the social brain in *children* is impacted by long-term patterns of parental care, particularly the experience of parent-child synchrony (Levy et al., 2017; Pratt et al., 2016, 2018), this is the first study that tests how the same parenting patterns impact the social brain in *mothers*. Furthermore, our findings show how activations in the maternal social brain shape the child's social competencies at the transition to adolescence (hypothesis 3). Finally, our study uniquely demonstrates that exposure to chronic stress impairs the neural basis of empathy in mothers and pinpoints such disruptions to the neural marker of mature empathy; gamma oscillations in the viceromotor cortex. Whereas mothers' rudimentary empathy sustained by sensory processing and lower-frequency oscillations (alpha and beta) was unaffected by trauma, the higher-order representations of empathy, supported by gamma activity in the viceromotor cortex, did not express in mothers raising children under chronic stress. According to a recent UNISEF report, one in five children globally is growing up in the context of ethnic, religious, national, or tribal conflicts, amounting to approximately 530 million children world-wide. Our study directs attention to the mothers of these children and underscores the need to develop targeted interventions for mothers who are raising children in the shadow of war and conflict.

### 4.1 Parent-child synchrony and maternal viceromotor gamma

The parent-child interface provides an evolutionary-salient context that sculpts both the parent and child's brain in profound ways (Feldman, 2016; Feldman et al., 2019). Changes in the maternal brain upon the birth of a child function to direct the mother's mental and physical resources, efforts, and attention to the child's well-being and to initiate the expression of well-adapted and synchronous parenting (Feldman, 2015c). Such synchronous parenting, once launched, has been shown in longitudinal studies to be individually stable across lengthy periods from infancy to adolescence (Feldman, 2010; Feldman et al., 2013) and the current findings similarly indicate stability in mother-child synchrony for over a decade. Studies in children indicate that the experience of synchrony in early childhood shapes the neural basis of attachment in adolescence (Pratt et al., 2018). Our results for mothers mirror the findings for children and show that parent-child synchrony - the partners' mutual adaptation to each other's affect, rhythms, and communication - shapes the mother's neural response, contributing to the expression of gamma rhythms in viceromotor cortex.

Studies in children (Pratt et al., 2018) and romantic couples (Kinreich et al., 2017) described associations between behavioral synchrony and gamma rhythms and the current findings show the same association in mothers. Moreover, gamma activity has been shown to increase in response to infant cues (Esposito et al., 2015), suggesting that gamma oscillations in human adults may function as a general caregiving-related signal. Gamma rhythms have been suggested to sustain bottom-up processes (Bastos et al., 2015) and, in combination with the current findings, these distinct lines of research point to the possibility that gamma may be an important neural rhythm for parenting. This suggestion is supported by previous evidence highlighting gamma's role in social and affective communication in general (Kinreich et al., 2017; Pratt et al., 2018; Symons et al., 2016), and during child-mother communication in particular (Levy et al., 2017). We propose that the role of gamma rhythms in parenting may be connected to the fact that gamma charts a developmental marker of maturity (Chang et al., 2016; Fabrizi et al., 2016; Levy et al., 2018; Uhlhaas et al., 2009), but this hypothesis requires much further research assessing gamma oscillations in mothers and fathers in comparison with non-parents, across stages of child development, and in various high-risk conditions related to parent (e.g., maternal depression), child (e.g., prematurity, ASD), or context (e.g., chronic stress, poverty).

### 4.2 Maternal neural response in viceromotor cortex, parenting, and chronic stress

Imaging studies of the parental brain using fMRI accord with our findings in describing the involvement of the ACC and AI, found here as part of the mother's empathy circuit, in the neural basis of parental attachment. In reviewing imaging studies of the parental brain, it was found that the ACC and AI showed activation in response to infant cues in all reviewed studies, regardless of whether the infant stimuli was auditory, visual, or multi-modal (Feldman, 2015c). Additionally, correlations were found between maternal sensitivity and neural activity: Mothers exhibiting more behavioral synchrony showed greater responses to their own infants' videos in the ACC (Atzil et al., 2011) and perception of mother-child synchrony activated maternal ACC (Atzil et al., 2014). A series of MEG studies showed modality-independent response to infant cues in the OFC in the first half-second, indexing an early mechanism that enables the parental brain to rapidly differentiate infant cues from other social signals (Kringelbach et al., 2008; Parsons et al., 2013; Young et al., 2016). Furthermore, mothers suffering from early-life maltreatment exhibited less sensitive behavior toward their children and also displayed abnormal activity in the insula and associated regions (Neukel et al., 2018). Thus, whereas our study uniquely assesses mothers of adolescents and link activations in the maternal brain to a decade of observed parenting, the results are consistent in several aspects with prior research, including the specific brain regions, oscillatory patterns, and associations with sensitive parenting and interactive synchrony found in prior studies.

Despite consistency with prior research highlighting the role of viceromotor gamma in mature empathy (Levy et al., 2018), our findings also present evidence that this neural signature is impaired when mothers must raise children in a context of chronic stress, results that have important implications for child and adolescent psychiatry. Our findings add to the growing knowledge on the impact of chronic stress and trauma on the mother's neurobiological systems, including salivary and hair cortisol, oxytocin, and immune biomarkers (Halevi et al., 2017; Ulmer-Yaniv et al., 2018). Such disruptions in the mother were found to impact the parallel systems in the child and, as mediated by caregiving patterns, and, consequently, to predict child psychopathology in the context of chronic early stress. Our findings showing an influence of the maternal brain on her child’s prosocial behavior are unique. Because the neural mechanisms underlying social relationships are not easily accessible, previous studies focused mainly on the influence of parental behavior, cognitions, and mental-health parameters on their children’s outcomes in the context of chronic early stress (e.g. Halevi et al., 2016, 2017). In addition to these psychological parameters, recent studies described several physiological pathways that mediate the effects of parental trauma exposure on children adaptation, including epigenetic, cardiovascular, immune, and hormonal systems (Ulmer-Yaniv et al., 2018). The current findings add the element of the maternal brain and show the impact of mother's brain activations on the child's well-being in the context of prolonged early stress.

### 4.3 Children’s prosocial abilities and chronic trauma

Prosocial abilities in children develop in the context of parental care and are related to synchrony and sensitive parenting (Feldman, 2007). Trauma has been previously shown to compromise synchrony and sensitive parenting (Feldman and Vengrober, 2011) and the current findings are consistent with these results. As such, it would be reasonable to expect that prosociality in the war-exposed group would be lower; however, our findings indicate no differences in prosociality between children who live in areas which are constantly under war and stressful life routines and those living in low-risk contexts. This, however, is not completely at odds with the literature: while some studies found that trauma and stress did impact child prosociality (Bergmann et al., 2016; Daud et al., 2008), others showed no differences (Flouri et al., 2010; Küenzlen et al., 2016). Interestingly, however, the studies which did report such effect implicated compromised parenting related to intra-familial factors (Bergmann et al., 2016; Daud et al., 2008) and not to external sources of trauma or stress. This may provide a possible explanation for why child prosociality in the current study was not directly affected by trauma, as our families suffered an external trauma and not trauma originating in the family (e.g., domestic violence). This interpretation is further strengthened by our findings that child prosociality was only affected by trauma via mother-child synchrony and the mother's neural empathic response. Future investigations are needed to test this new hypothesis, comparing child prosociality in ELS contexts stemming from external versus intra-familial sources.

### 4.4 Broader implications

Overall, parental stress and PTSD were found to increase rates of depression, anxiety, internalization and externalization problems in offspring, regardless of the child’s trauma exposure (Leen-Feldner et al., 2013). Parents exposed to trauma report less satisfaction from parenting, lower relationship quality with their children, and greater conflict, aggression, hostility, anger, and disengagement from their children. In previous assessments of this cohort, we found that war-exposed mothers were less sensitive and empathic and more stressed and negative (Halevi et al., 2017). Our results validate these behavioral findings from a neural perspective, implicating the brain substrate associated with maturity and empathy as a mediator between maternal behavior and children outcomes. Importantly, war-exposed and control children did not differ in overall prosociality, which was predicted by the quality of caregiving.

Our study is among the handful of studies showing prediction from activations of the parental brain, typically in infancy, to children's social and mental health outcomes and is the first to test this in adolescence. Children's social abilities index an important resilience component, particularly in the context of trauma (Halevi et al., 2017; Ulmer-Yaniv et al., 2018) and have been shown to predict lower externalizing behavior across adolescence and greater academic achievement and peer social preference (Caprara et al., 2000). Our study, therefore, suggests a possible role for the maternal brain and its contribution to shaping children's social outcome via parenting. These findings point to a possible transgenerational transmission of empathy (Vogel, 1994); when the neural underpinnings of empathy are impaired in the mother this mediates compromise in the child's empathic abilities via the decrease in the quality of caregiving.

Our findings suggest that the quality of parenting, as indexed by mother-child synchrony, is a crucial rearing component throughout childhood and is predictive of mothers’ empathic abilities. The ability to synchronize with one's child reflects dyadic reciprocity, regulation/adaptation, interactive fluency, behavioral empathy, supportive presence, positive affect, recognition, expansion, containment and appropriate expression. The present findings therefore consolidate an important correspondence between these interactive abilities and empathy at the intersection of behavior and brain. This interesting observation has been observed in a similar way in healthy mothers and their children (Levy et al., 2017) as well as in the context of intergroup relations (Levy et al., 2016). Hence, we propose that the neural index of empathy may be implemented in various contexts (e.g., interventions, intergroup relations) to evaluate social interactions. Furthermore, we found here that this important parental component is highly sensitive to trauma and adverse life conditions; trauma undermines this component, possibly acting as a mediator to the impairment of maternal neural empathic response, which in turn, reduces child prosociality. We would like to suggest that this directional pattern (mother-child synchrony → mother neural empathy → child prosociality) may have implications for interventions by highlighting the importance of supporting mothers in contexts of high stress, particularly war-related stress. Our findings highlight the need to pay attention to the mother side of the parent-child dyad, not only to the child's perspective, and to devise special interventions that empower mothers, bolster synchrony, and enhance maternal empathy when mothers must raise children in contexts of continuous fear, chronic stress, unpredictable war, or repeated trauma. Such interventions may result in improving not only each individual's prosocial faculties, but also the quality of their relationship.

## Conflict of interest

None.

## Footnotes

1 Children’s brain was also imaged and results will be presented elsewhere.
